# Hibiscus Anthocyanins Extracts Induce Apoptosis by Activating AMP-Activated Protein Kinase in Human Colorectal Cancer Cells

**DOI:** 10.3390/nu15183972

**Published:** 2023-09-14

**Authors:** Ming-Chang Tsai, Ching-Chun Chen, Tsui-Hwa Tseng, Yun-Ching Chang, Yi-Jie Lin, I-Ning Tsai, Chi-Chih Wang, Chau-Jong Wang

**Affiliations:** 1 Division of Gastroenterology and Hepatology, Department of Internal Medicine, Chung Shan Medical University Hospital, Taichung 402, Taiwan; tsaimc1110@gmail.com; 2 School of Medical, Chung Shan Medical University, Taichung 402, Taiwan; 3 Institute of Medicine, Chung Shan Medical University, Taichung 402, Taiwan; sandy985619@gmail.com (C.-C.C.); 0312elise@gmail.com (I.-N.T.); 4 Department of Medical Applied Chemistry, Chung Shan Medical University, Taichung 402, Taiwan; tht@csmu.edu.tw; 5 Department of Health Industry Technology Management, Chung Shan Medical University, Taichung 402, Taiwan; changyc@csmu.edu.tw (Y.-C.C.); exist7730@gmail.com (Y.-J.L.); 6 Department of Medical Research, Chung Shan Medical University Hospital, Taichung 402, Taiwan

**Keywords:** Hibiscus anthocyanidin extract, apoptosis, AMPK, Fas/Fas L, colorectal cancer

## Abstract

Apoptosis, a programmed cell death process preventing cancer development, can be evaded by cancer cells. AMP-activated protein kinase (AMPK) regulates energy levels and is a key research topic in cancer prevention and treatment. Some bioactive components of *Hibiscus sabdariffa* L. (HAs), including anthocyanins, have potential anticancer properties. Our study investigated the in vitro cytotoxic potential and mode of action of HAs extracts containing anthocyanins in colorectal cancer cells. The results showed that Hibiscus anthocyanin-rich extracts induced apoptosis in human colorectal cancer cells through the activation of multiple signaling pathways of AMPK. We observed the dose–response and time-dependent induction of apoptosis with HAs. Subsequently, the activation of Fas-mediated proteins triggered apoptotic pathways associated with Fas-mediated apoptosis-related proteins, including caspase-8/tBid. This caused the release of cytochrome C from the mitochondria, resulting in caspase-3 cleavage and apoptosis activation in intestinal cancer cells. These data elucidate the relationship between Has’ regulation of apoptosis-related proteins in colorectal cancer cells and apoptotic pathways.

## 1. Introduction

Apoptosis is a programmed cell death process regulated through caspase activation to maintain normal cell populations. When cellular abnormalities arise, damaged cells undergo apoptosis, which prevents cancer development. However, cancer cells can avoid apoptosis and continuously divide. Most anticancer therapies trigger apoptosis induction to eliminate malignant cells. Apoptosis can be activated through the death receptor signal pathway or intrinsic apoptosis pathway wherein changes in the integrity of the mitochondrial membrane are regulated by Bcl-2 family proteins [1]. A crosstalk of these two major apoptotic pathways has also been identified [2,3]. Researchers have expressed interest in natural products that can modulate various cancer cell death signaling pathways and have investigated their modes of action against cancers.

AMP-activated protein kinase (AMPK) is a crucial mediator in the maintenance of cellular energy homeostasis. AMPK, which regulates metabolic energy, is a common research focus for the treatment of metabolic syndromes such as type-2 diabetes. Metformin is an AMPK activator that reduces the incidence of cancer [4,5]. The deregulation of cellular energetics is a hallmark of cancer. Activated AMPK may influence many effector proteins involved in the regulatory processes and pathogenesis of cancers. AMPK activation may suppress metabolic tumor growth through the regulation of energy levels, enforcement of metabolic checkpoints, and inhibition of cell growth. Thus, AMPKs have been widely investigated in studies on cancer prevention and treatment [6].

Colorectal cancer (CRC) is the most prevalent form of cancer in Taiwan. The incidence and mortality rates of CRC have increased, particularly among young adults [7,8], although treatment quality has improved. CRC therapy includes radiotherapy, chemotherapy, and surgery. After these treatments, delaying the onset and progression of CRC is the primary focus of doctors. CRC development is influenced by genetic, environmental, and nutritional factors, with nutritional factors playing both protective and causal roles. Certain health foods and beverages may contain components exhibiting antiproliferative effects on colorectal cancer cells [9,10,11,12,13]. *Hibiscus sabdariffa* L. (HAs), also known as Roselle, belongs to the Malvaceae family. Widely distributed and cultivated in tropical and subtropical regions, the flowering plant is used in cosmetics, food, and medicine. Its main bioactive components are anthocyanins, phenolic acids, polysaccharides, and flavonoids [14,15]. Many in vitro and in vivo studies have demonstrated the beneficial pharmacological properties and functions of anthocyanin extracts, including hepatoprotection, antioxidant activity, anti-atherosclerotic effects, and anti-inflammation and anticancer properties. Several previous research studies have explored the relationship between anthocyanins and cancer, specifically their role in inducing apoptosis in cancer cells. Some key findings include anticancer properties of berry anthocyanins, apoptotic effects of grape anthocyanins, the connection between blueberry anthocyanins and prostate cancer, etc. [16,17,18]. In addition, our previous studies showed that Hibiscus anthocyanins may protect against cancer by inhibiting the growth, proliferation, and migration of cancer cells and by reducing inflammation [19,20,21,22]. However, the extract’s relationship with CRC remains unclear. This study analyzed the cytotoxic potential and mode of action of the anthocyanins from HAs in vitro.

## 2. Materials and Methods

### 2.1. Reagents and Chemicals

Dulbecco’s Modified Eagle’s Medium (DMEM) and fetal bovine serum (FBS) were purchased from Gibco Ltd. (Grand Island, NY, USA). Antibodies for the testing of p-AMPK (#2535), AMPK (#2532), and p-Akt (#9271) were obtained from Cell Signaling Technology (Danvers, MA, USA). Other antibodies, including Bcl-2 family AIF (sc-13116), Bad (sc-8044), Bax (sc-526), Bcl-xl (sc-8392), Bid (sc-6538), Cytochrome C (sc-13560) and PARP (sc-377015), caspases-3 (sc-7148), caspases-8 (sc-7890), caspases-9 (sc-73548), and Fas/FasL (sc-7886, sc-6237), were acquired from Santa Cruz (Santa Cruz, CA, USA). Antibiotic-antimycotic (100X) and trypsin-EDTA were purchased from Gibco Ltd. (Grand Island, NY, USA). 4,6-diamidino-2-phenylindole (DAPI), Tris base, propidium iodide (PI), and other materials were acquired from Sigma Chemical (St. Louis, MO, USA).

### 2.2. Preparation of HAs

First, extraction was performed on Hibiscus flowers [22]; specifically, 20 g of dried Hibiscus flower was combined with methanol and 1% HCl for 1 day at 4 °C. The extract was filtered and concentrated; subsequently, the collected precipitate was loaded into an Amberlife Diaion HP-20 resin column and allowed to settle for 24 h. Thereafter, we cleaned the column with distilled water containing 0.1% HCl solution and used methanol to elute. The filtrate was collected and subsequently lyophilized to obtain approximately 2 g of HAs, which was stored at −20 °C before use. 100 mg/mL HAs stock solution in ddH_2_O water was prepared and stored at 4 °C, protected from light.

### 2.3. HPLC Analysis

Total HAs were extracted with the Fuleki and Francis method [23]. In particular, a 100 μL aliquot of HAs (10 mg/mL) was diluted with 3 mL of pH 1.0 and pH 4.5 buffers. The optical density (OD) of the sample was measured at 535 nm, using distilled water as a reference. The change in OD was calculated by subtracting the total OD at pH 4.5 from the total OD at pH 1.0. Both values were calculated with OD readings and standard dilution and calculation factors. For HAs standardization, cyanidin and delphinidin parameters were determined with high-performance liquid chromatography (HPLC) using the symmetry shield RP18 column (3.5 μm, 4.6 × 150 mm) and UV–VIS detector (monitored at 530 nm). The mobile phase comprised H_2_O, 10% formic acid, and methanol (65/35, *v*/*v*.). We used 1 mL acidic methanol (HCl:CH_3_OH = 1:1, *v*/*v*.) to dissolve 1 mg sample and boiled it at 95 °C for 30 min. Subsequently, 10 μL of the solution was injected into a chromatography column, and the flow rate was maintained at 1 mL/min. The result was appraised with cyanidin and delphinidin.

### 2.4. Cell Culture

The human colon cancer cell line LoVo was obtained from the American Tissue Culture Collection. The LoVo cell line was cultured with DMEM complemented with 10% FBS, 2 mmol/L glutamine, 100 μg/mL antibiotic-antimycotic (100X), and 1 mmol/L HEPES buffer in an incubator with 5% CO_2_ at 37 °C.

### 2.5. MTT Assay

For the MTT assay, we first used 3-[4,5-dimethylthiazol-2-yl]-2,5-diphenyltetrazolium bromide (MTT), a yellow water-soluble solid that can be metabolized by dehydrogenase in mitochondria in cells and cut off at the tetrazolium ring. The purple insoluble precipitate formazan (3-[4,5-dimethylthiazol-2-yl]-2,5-diphenyl-formazan) accumulated in the cells and subsequently dissolved in an organic solution. Its absorbance level was measured to be 563 nm (OD). LoVo cells were seeded in a culture dish overnight, and we treated them with drugs (0, 1, 2, 3 mg/mL) for a specified duration of 0, 12, 24, 36, and 48 h. Subsequently, the medium was aspirated, and another medium containing MTT was added for a two-hour incubation period. After removing the medium containing MTT, we dissolved the purple crystals with DMSO, and the optical density was measured to be 563 nm.

### 2.6. DAPI Staining for Cell Apoptosis

DAPI is a blue fluorescent dye that can penetrate cell membranes. DAPI fluorescent staining was used to observe chromatin changes during cell apoptosis. Human LoVo cancer cells (106 cells/mL) were treated with 1, 2, and 3 mg/mL HAs for 24 and 48 h. Post treatment, the cell monolayers were rinsed in PBS and fixed with 4% paraformaldehyde for 30 min at room temperature. After fixing, we prepared 0.2% Triton X-100 in PBS to treat the cells and permeabilized them three times. Subsequently, they were incubated with DAPI for 30 min and subjected to three additional PBS washes. The apoptotic nuclei (intensely stained with a fragmented nucleus and condensed chromatin) were examined and photographed under 200× magnification using a fluorescent microscope with a 340/380 nm excitation filter.

### 2.7. Flow Cytometry for Sub-G1 Phase

The DNA content of HAs-treated cells (3 mg/mL, 0–48 h) was determined with a Becton Dickinson flow cytometer with PI staining. The DNA content distribution was expressed as sub-G1, G0/G1, S, and G2/M phases. The percentage of hypodiploid cells (sub-G1) over the total cells was calculated and expressed in terms of the percent of apoptosis.

### 2.8. Measurement of Mitochondrial Membrane Potential

Mitochondrial membrane potential was assessed with JC-1 (C_25_H_27_C_l4_IN_4)_. LoVo Cells (1 × 10^6^) were treated with HAs (1, 2, and 3 mg/mL) for 24 h, then subsequently harvested and washed with cold PBS twice. The cells were incubated with the fresh culture medium containing 2.5 μg/mL of JC-1 dye for 30 min at 37 °C. Cells were collected using centrifugation at 2000× *g* for 5 min and subsequently washed once with DMEM to enable observation. Red and green fluorescence emissions were photographed under fluorescence microscopy. Additionally, the cell was analyzed with flow cytometry using an excitation wavelength of 488 nm and an emission wavelength of 530 nm (green fluorescence). An increase in green fluorescent (FI) intensity represented mitochondrial swelling and loss of mitochondrial membrane potential. Quantitative analysis of the mitochondrial membrane potential was performed with a flow cytometer (FACS).

### 2.9. Western Blot Assay

After HAs treatment, total cell lysates were split into equal proteins in polyacrylamide gel (8–12%) and transferred to PVDF transfer membranes. Next, the blot membrane was washed with PBS three times after steeping in blocking buffer (PBS with 5% nonfat milk) for 1–3 h and incubated in primary antibody buffer at 4 °C overnight. Subsequently, the blot was washed with PBS three times and incubated in a secondary antibody buffer at 4 °C for 1 h. Lastly, the ECL detection system revealed the antigen-antibody complex. The relative band density of the image was quantitated with a densitometer.

### 2.10. Statistical Analysis

Data are reported as the means ± standard deviation of three independent experiments, and the groups were compared with one-way analysis of variance. Differences were considered significant at *p* < 0.05.

## 3. Results

### 3.1. Component Analysis

**Figure 1:**
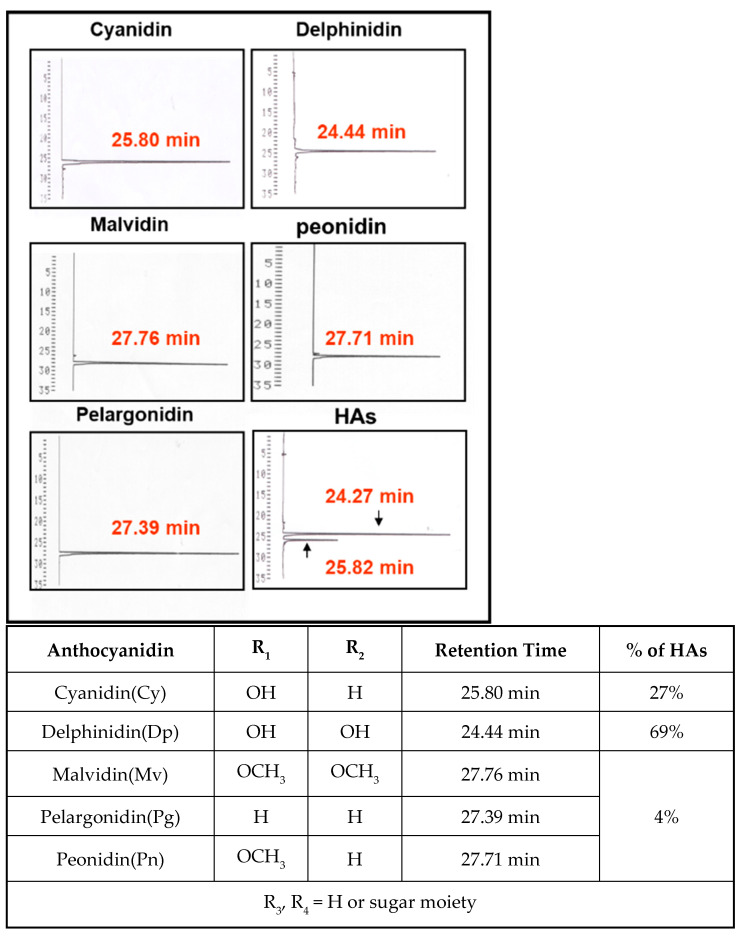
The HPLC chromatogram of HAs. Arrow indicates peaks of delphinidin and cyanidin.

First, HPLC was used to detect the major components of hibiscus anthocyanins. The results of Figure 1 indicate that the peak of HAs most closely matched the retention time of standard cyanidin and delphinidin. The contents of cyanidin and delphinidin in HAs were 27% and 69%, respectively. These findings verified that cyanidin and delphinidin are the two primary components of HAs.

### 3.2. HAs Inducing Cytotoxicity Apoptosis

**Figure 2:**
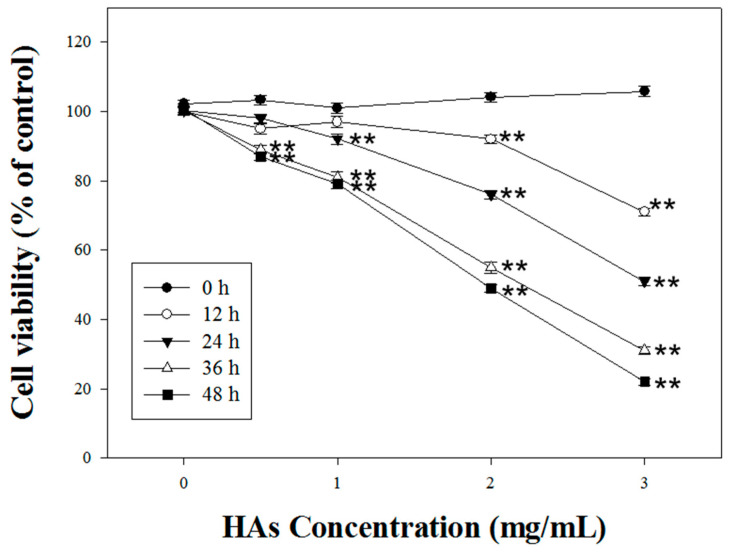
The cytotoxic effect of HAs on LoVo cells. The cells were treated with 0, 1, 2, and 3 mg/mL HAs for 0, 12, 24, 36, and 48 h and cell viability was measured with MTT assay. Data are shown as mean ± SD. Results were analyzed with Student’s t-test. ** *p* < 0.005.

We investigated HAs’ modulation of the cell viability of LoVo colorectal cancer cells. HAs with various concentrations (0, 1, 2, and 3 mg/mL) were administered over 0, 12, 24, and 48 h. HAs with the highest concentration (3 mg/mL) reached the IC_50_ after treatment for 24 h (Figure 2).

**Figure 3:**
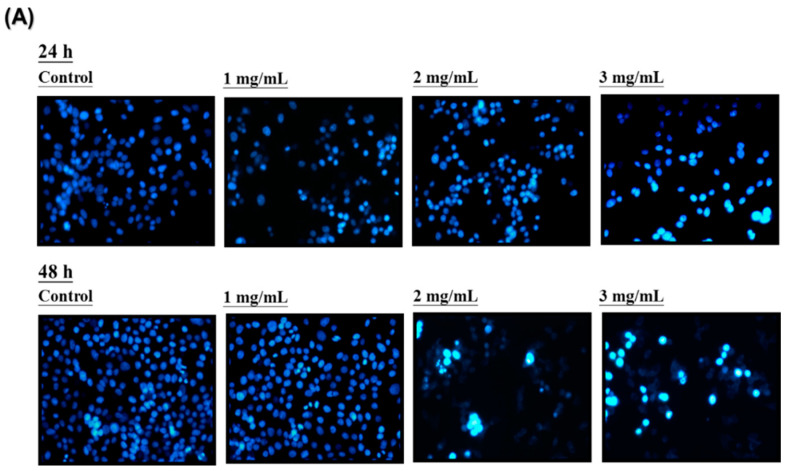

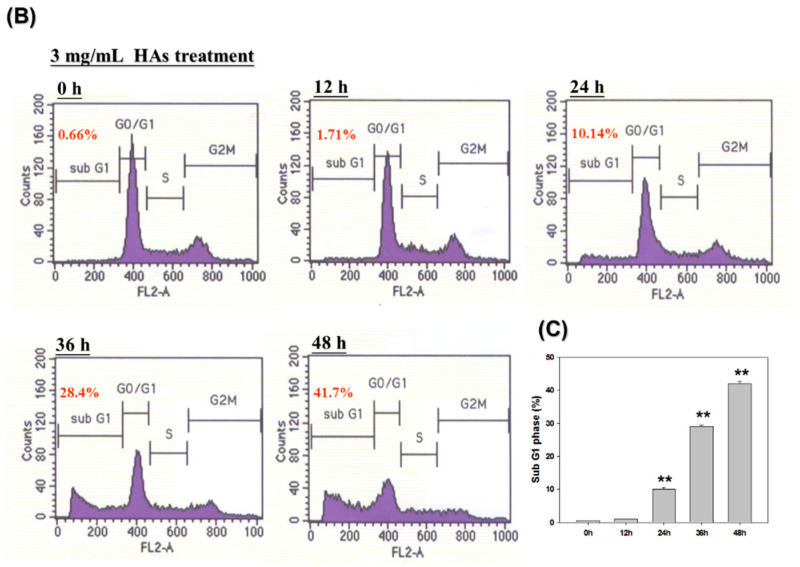
Apoptosis induction in HAs-treated LoVo cells. (**A**) LoVo cells treated with HAs under various concentrations (0, 1, 2, and 3 mg/mL) for 24 or 48 h. Apoptosis cells assayed by DAPI staining and observed with fluorescence microscopy (magnification 200×). (**B**) LoVo cells treated with HAs of 3 mg/mL for 0, 12, 24, 36, and 48 h. Cell apoptosis detected through PI staining and analyzed with flow cytometry. (**C**) DNA content in sub-G1 cell phase. Data shown as mean ± SD. Results analyzed with Student t test. ** *p* < 0.005.

LoVo cells were cultured at 37 °C with various concentrations (0, 1, 2, and 3 mg/mL) of HAs. After 24 h and 48 h of action, the cell type and survival status were observed with an optical microscope and DAPI staining, respectively. Figure 3A indicates a negative correlation between HAs concentration and cell survival rate. However, higher HAs concentrations also increased cell shrinkage and apoptotic body generation. DAPI staining results indicated apoptosis through dense staining of the nucleus. Pro-apoptotic body presence was observed with flow cytometry to verify HAs causing apoptosis in LoVo cells. The cells were treated with the highest concentration (3 mg/mL) of HAs and LoVo cells were incubated at 37 °C. Figure 3B demonstrates that at various points in time (0, 12, 24, 36, and 48 h), the cells cultured for 48 h produced a substantial amount of pro-apoptotic bodies. DNA content during the sub-G1 phase of cells increased from 0.66% to 41.7%, representing an approximate 40% increase. These results indicate a time-dependent phenomenon (Figure 3C).

### 3.3. Effects of HAs on the Mitochondrial Membrane Potential

**Figure 4:**
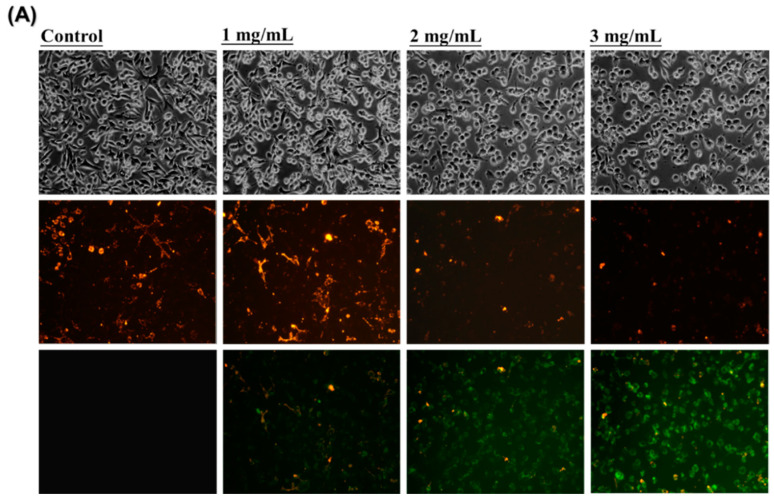

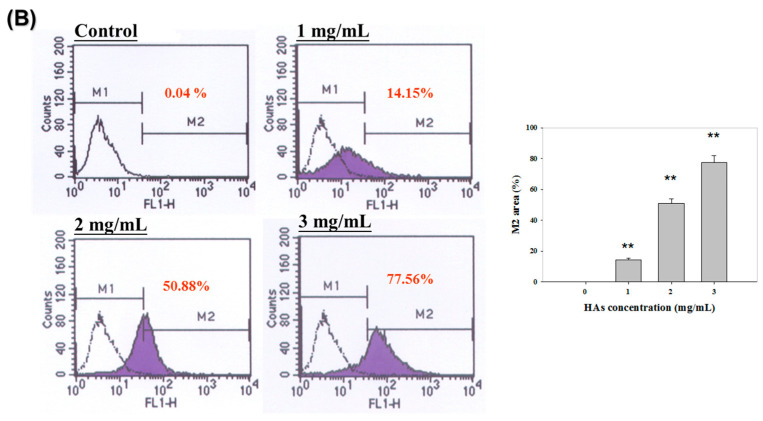
Mitochondria membrane potential (ΔΨm) assessment of LoVo cells treated with HAs. (**A**) LoVo cells treated with HAs (1, 2, and 3 mg/mL) for 24 h; cells stained with JC-1. Monomeric form JC-1 with green fluorescence indicated the dissipation of MMP. (**B**) LoVo cells detected with flow cytometry. M2 area percentage represents the potential of mitochondrial membrane depolarization. Values are the mean SD (*n* = 3). ** *p* < 0.005, compared with the control group (0 mg/mL) of the HAs concentration, respectively.

LoVo colorectal cancer cells were treated with HAs at different concentrations (0, 1, 2, and 3 mg/mL). When JC-1 staining was conducted, LoVo cells not treated with HAs exhibited red fluorescence (Figure 4A). When Has concentration increased, the red fluorescence gradually transitioned to green fluorescence. Furthermore, the dye intensity of JC-1 was analyzed with flow cytometry to assess mitochondrial membrane potentials. The green absorption intensity of JC-1 dye was compared with that in the control group. Green fluorescence intensity increased from 0.04% to 77.56%. Treatment of LoVo cells with HAs resulted in the damage and disintegration of mitochondria in a dose-dependent manner. These results suggest that HAs treatment may induce a cell death program in colorectal cancer cells through the intrinsic apoptotic pathway.

### 3.4. The Expression of HAs on the Apoptosis-Associated Proteins of LoVo Cells

**Figure 5:**
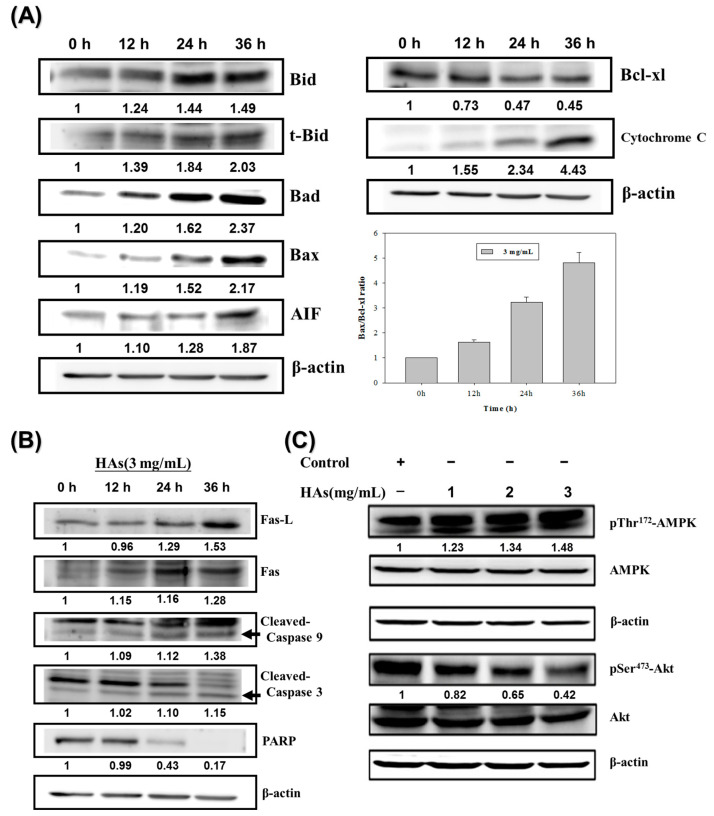
Effects of HAs on apoptosis-associated proteins in LoVo cells. (**A**) LoVo cells treated with HAs (3 mg/mL) for 24–48 h. Cells subjected to Western blotting to analyze signaling pathways related to mitochondria membrane potential. (**B**) Signaling pathways related to apoptosis receptors. (**C**) LoVo cells treated with 1, 2, and 3 mg/mL HAs for 24 h. Cell expression and phosphorylation of AMPK and Akt proteins were analyzed using Western blotting.

To investigate the mechanism underlying HAs-induced apoptosis in LoVo colorectal cancer cells, the Western immunoblotting method was employed. First, LoVo cells were treated with the highest concentration of HAs (3 mg/mL) at 37 °C for 0, 12, 24, and 36 h to assess the expression of apoptosis-related proteins. The results indicated that HAs elevated the expression of tBid, Bax, and Bad, triggering a cascade of mitochondrial death pathways. This process mediated an upregulation in the expression of the downstream protein apoptosis-inducing factor (AIF), culminating in cell apoptosis (Figure 5A). Bcl-2 family members that inhibit apoptosis, such as Bcl-xl, significantly decreased. Treatment with HAs induced a time-dependent increase in the level of cytochrome C and increased the expression of Fas/Fas L, which both activate the extrinsic apoptosis pathway (Figure 5B). Additionally, HAs triggered the activation of multiple members of the caspase family, namely caspase 3, 8, 9, and PARP involved in the DNA repair function (Figure 5B). Lastly, AMP-activated protein kinase (AMPK) includes the highly conserved serine/threonine protein kinase and may regulate cancer cell metabolic energy. Figure 5C indicates that increasing HAs concentration promoted p-AMPK expression and inhibited p-Akt expression. Therefore, HAs may regulate proteins associated with apoptotic pathways to induce apoptosis.

**Figure 6:**
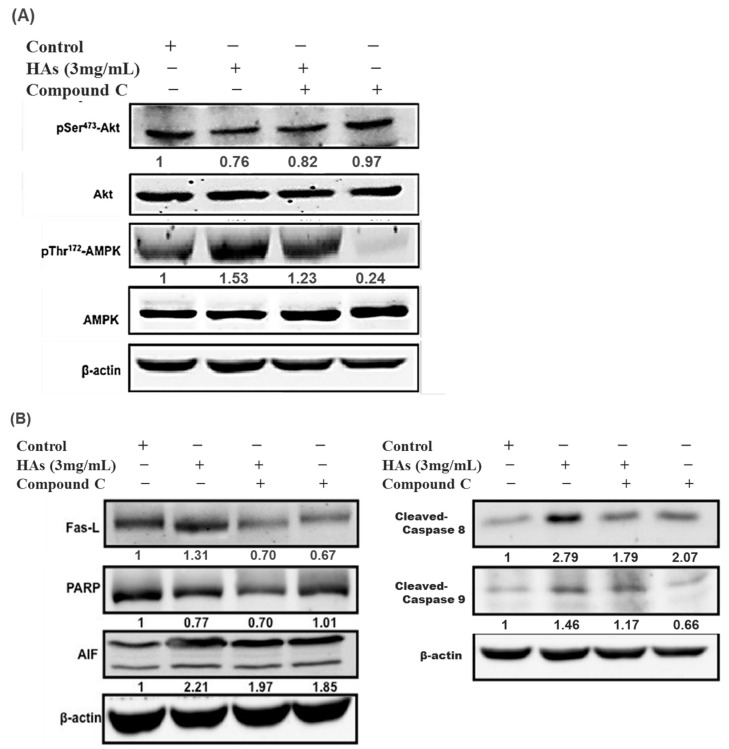
HAs-regulated expression of apoptotic proteins through the activation of the AMPK signaling pathway in LoVo cells. (**A**) LoVo cells treated with Compound C (10 μM) for 1 h and subsequently treated with HAs (3 mg/mL) for 24 h. Treated cells were subjected to Western blotting to analyze the phosphorylation of AMPK and Akt. (**B**) AMPK activating apoptotic proteins.

To elucidate why HAs triggered the apoptosis of LoVo colon cells, Compound C (an AMPK inhibitor) was added to the solution. The LoVo cells were treated with the highest concentration of HAs (3 mg/mL) at 37 °C for 24 h. Compound C (10 μM) was added one h before HAs to inhibit p-AMPK and verify the presumed relationships of other proteins. As illustrated in Figure 6A, the simultaneous addition of Compound C and HAs was associated with a lower p-AMPK expression compared with the use of HAs alone. Additionally, the p-AMPK expression significantly decreased when Compound C was used independently. These results demonstrate that HAs activated AMPK. Figure 6B indicates that p-Akt expression levels increased after p-AMPK inhibition, providing evidence that p-AMPK acts as an upstream regulator of p-Akt/Akt. Additionally, Figure 6B indicates that Compound C affected the expression of FasL and downstream proteins, including caspase, PARP, and AIF. This suggests that HAs modulated the exogenous and endogenous apoptosis pathways in LoVo cancer cells through the p-AMPK/FasL pathway to impede cell growth.

## 4. Discussion

Hibiscus extracts that are rich in anthocyanins exhibit a wide range of biological activities, including anti-apoptosis, anti-angiogenesis [14], antioxidation [24,25,26], anticancer, and anti-metastasis [21] in human cancer cells and animal models. Apoptosis is induced in cancer cells through the activation of extrinsic and intrinsic signaling pathways. AMPK and Akt proteins are pivotal in the apoptotic pathway, underscoring their relevance in cancer treatment [27,28]. Akt is a regulator of various biological functions closely related to cell growth, proliferation, and apoptosis. Our findings indicate that HAs upregulated the protein expression of AMPK and inhibited the phosphorylation of Akt protein, thereby inducing apoptosis in cells. AMPK is composed of trimeric protein polymers and is central to protein synthesis, cell growth, and apoptosis [29]. The experimental results indicated that AMPK protein expression increased the protein content of the death receptor Fas/Fas ligand on the cell membrane (Figure 5). The combination of Fas and Fas L caused FADD (Fas-associated protein with death domain) recruitment and death-inducing signaling complex (DISC) formation. This facilitated the binding of procaspase-8 to DISC, thereby activating caspase-8 and its downstream proteins and promoting a cascade of apoptosis reactions in cells (Figure 3).

Apoptosis is closely related to mitochondrial membrane integrity [30]. The cytochrome C and AIF were estimated using the lipophilic cationic probe JC-1. The results demonstrated the breakdown of the mitochondrial membrane potential (MMP), which occurs through the apoptotic pathway. The Bcl-2 family exerts its influence on the intrinsic apoptotic pathway by regulating the interplay between proapoptotic proteins and antiapoptotic proteins, thereby affecting the cell’s commitment to the apoptotic pathway [31,32]. The BH3-only proapoptotic Bcl-2 members, namely Bax, Bad, and tBid, are antagonized by anti-apoptotic family members, including Bcl-xl [33]. Bcl-xl promotes cell survival through the maintenance of the integrity of the outer mitochondrial membrane and the prevention of the release of cytochrome c from mitochondria [34]. Caspases are a family of cysteine proteases that act as mediators of apoptosis and promote apoptotic morphology through the cleaving of various cellular substrates [35]. The intrinsic pathway of apoptosis involves the activation of caspase-9, which cleaves and activates caspase-3 [36]. Our experiments found that HAs enhanced the expression of pro-apoptotic members and decreased the expression of anti-apoptotic members to cause disruption of the MMP (Figure 4 and Figure 5).

Cancer cells often have reduced mitochondrial oxidative phosphorylation, increased glycolysis, and altered metabolism of energy. AMPK activation can promote mitochondrial biogenesis, inhibit glycolysis, and restore energy balance in cancer cells, thereby regulating cell survival signaling pathways such as the Akt signaling pathway. Previous studies have indicated that natural compounds, including curcumin, gallocatechin, and paclitaxel, have been proven to be effective in treating breast cancer by inducing apoptosis [37]. Quercetin has also been shown to induce apoptosis in bladder cancer cells [38,39], while Baicalein induces apoptosis in human lung carcinoma A549 cells [40], all through the AMPK pathway. Therefore, we used Compound C to inhibit AMPK, which revealed that HAs induce apoptosis via the AMPK pathway and the apoptosis signaling pathway, which is triggered by Fas/Fas L. Figure 6 indicates that adding AMPK inhibitors increased the expression of p-Akt/Akt and significantly downregulated apoptosis-related proteins such as Fas L, PARP, AIF, and caspase-9, suggesting that HAs induced apoptosis of colorectal cancer LoVo cells through exogenous and intrinsic pathways. The inhibition of AMPK by Compound C highlights the inhibitor’s critical role in mediating the pro-apoptotic effects of HAs. These findings reveal the underlying mechanism through which HAs affect cancer cell death and provide a potential avenue for therapy aimed at promoting cancer cell apoptosis through the manipulation of the AMPK pathway. Delphinidin, a compound found in anthocyanins, has declared protection against various chronic conditions, consisting of cancer, cardiovascular disease, and diabetes, by activating AMPK in vitro and in vivo studies. Delphinidin also regulates downstream targets in multiple cancer cell lines, including breast, colon, prostate, and liver cancer, thereby impeding cancer cell proliferation, inducing apoptosis, and suppressing tumor growth [41]. Previous studies have indicated that anthocyanins such as delphinidin may bind to enzymes and regulate their activity. One notable example is the binding of delphinidin to angiotensin-converting enzyme (ACE), enabling it to regulate ACE activity [15].

**Figure 7:**
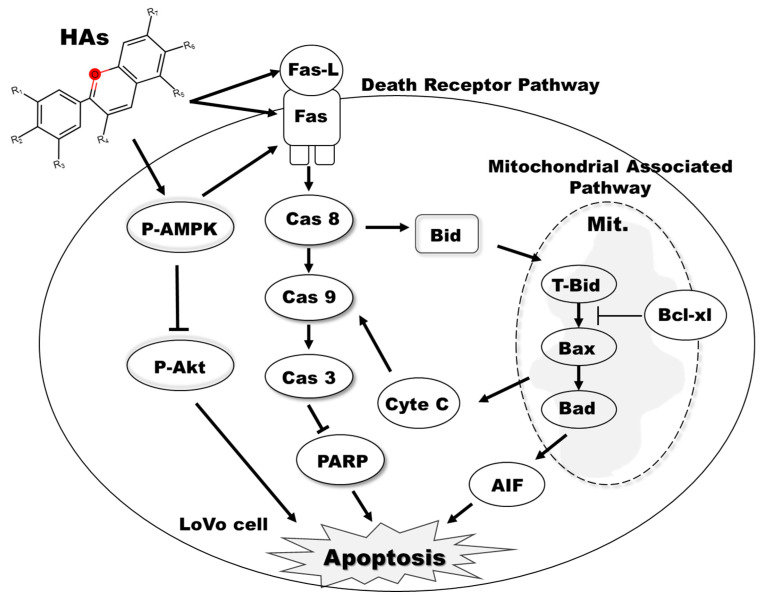
HAs induced LoVo cell apoptosis and inhibited the phosphorylation of Akt. AMPK activation increased expressions of Fas/Fas L through intrinsic and extrinsic apoptosis pathways.

Further investigations are required to ascertain whether our experimental findings stem from the binding of delphinidin to AMPK kinase. Findings in the literature indicate that HAs are rich in polyphenols, flavonoids, vitamin C, and other active compounds that impart significant antioxidant, anti-inflammatory, and anticancer properties. However, despite the multifunctional and pharmacological characteristics of HAs, the anti-colorectal cancer effects are still unclear. Our study provides evidence that HAs inhibit the AMPK and Akt signaling pathways to promote apoptosis in colorectal cancer cells. Several mechanisms contribute to these effects, including the activation of the Fas protein, subsequent activation of Fas-mediated apoptosis-related protein pathways (such as the caspase-8/tBid pathway), modulation of the mitochondria membrane potential, and subsequent release of cytochrome C from mitochondria. These effects suggest that the Hibiscus anthocyanidin extract can prevent and inhibit colorectal cancer by activating AMPK, inhibiting Akt, and increasing Fas/Fas L to produce intrinsic and extrinsic apoptosis. (Figure 7).

## Data Availability

Not applicable.
